# Marathon Performance Depends on Pacing Oscillations between Non Symmetric Extreme Values

**DOI:** 10.3390/ijerph19042463

**Published:** 2022-02-21

**Authors:** Jean-Renaud Pycke, Véronique Billat

**Affiliations:** 1 UMR8071—CNRS-Laboratoire de Mathématiques et Modélisation d’Evry (LaMME), University of Paris-Saclay (Evry), 91037 Evry, France; 2 Department of STAPS, University of Paris-Saclay (Evry), 91037 Evry, France; veronique.billat@univ-evry.fr

**Keywords:** extreme values, symmetry breaking, pacing strategy, optimization

## Abstract

A marathon was recently run in less than 2 h by a man who ran the three fastest marathons ever recorded in a span of three years—Eliud Kipchoge—in the Tokyo Olympic games. Here, we demonstrate that the best marathons were run according to a pace distribution that is statistically not constant and with negative asymmetry. The concept of mirror race enables us to show that the sign of asymmetry is not due to sampling fluctuations. We show that marathon performance depends on pacing oscillations between extreme values, and that even the best marathons ever run differ and can be improved upon. The utilization of extreme values and oscillations allows for recovery and optimization of the complementary aerobic and anaerobic metabolisms. Our findings suggest new ways to approach the pacing for optimizing endurance performance.

## 1. Introduction

For centuries, the limits of physiology and athletic records have fascinated scientists. In 2019, the sub-two-hour marathon barrier was broken with a time of 1:59:40.2. In 1925, Nobel laureate Archibald Vivian (AV) Hill published “The Physiological Basis of Athletic Records” [1].

Nowadays, one century later, advances in wearable sensor technology have enabled real-time measurement of physiological data during exercise [2]. Future directions in training are going to be about encouraging marathon runners. To achieve this, we must provide ways to improve satisfaction regarding training progress and with the feeling that it has been optimized. The results show that according to our hypothesis, we are testing the hypothesis that the ideal race, with the world’s best marathon runners, can have several degrees of optimization. This can be observed in Kipchoge’s last five marathons, since Kipchoge ran the three fastest marathons at the time across a span of three years, and even winning Olympic gold medals in the process (2016, 2021).

Hill’s and Kennelly’s approach to running the fastest marathon was based on the constant pace paradigm.

In Hill’s reference to Kennelly’s 1906 paper, he stated that the ideal way to run a race was not necessarily to win, but to achieve a new athletic record for the distance, and to do it by running it at a constant speed. Fifty years later, the physicist Joseph Keller modelled the predominating view that a runner should maintain a constant pace to achieve the shortest time in his paper: “A Theory of Competitive Running“ [3]. To determine the optimal race strategy, he used simple physics and mathematics to correlate the physiological attributes of runners with world track records. He derived the optimal speed variation t↦v(t) by formulating and solving a problem in the optimal control theory. For distances greater than 291 m, his theory predicted a maximum acceleration for 1 or 2 s, then constant speed throughout the race, until the final 1 or 2 s, and finally, a slight slowing down. At the time, his results confirmed the recognized view that a runner should maintain a constant speed to achieve the shortest time (Keller, 1973). Importantly, Keller admits his theory omitted several important variables, such as the up-and-down motion of the limbs, internal and external resistance, the depletion of fuel, and the accumulation and removal of waste products. These ideas were perhaps valid in 1925, when the fastest marathon time was only 2 h 29 min (by Albert Michelson, an average speed of 16.99 km/h, Port Chester, NY, USA).

In the same way, Arthur Kennelly desired to measure all points of a race. Delving deeper into Kennelly’s paper: “It is to be noted that all these speeds are average speeds over the courses. There is no evidence among the records to show what the speed was at different points in the course. So far, as concerns anything appearing in the data, the speed of a runner, for example, which averages 7.17 ms per s over a 1 km course, might be 10 m per s in the first part and 5 in the last part, or vice versa. Evidence is lacking to show what the facts are, and they are of great importance to the science of athletics. The speed of a world’s record type of trained runner might be determined at any or all points of a course, either by securing a light recording chronograph on the back of his belt, with a thread paid out as he ran, or by pacing the runner with a light motor-car carrying an automatic speed“ [4], p. 328. Nowadays, thanks to microchip technology, it is possible to measure every point during a marathon.

Physiologic processes are inherently never constant. French physiologist Claude Bernard wrote, in his classic book [5], that: “The use of averages in physiology and medicine most often gives only a false precision to the results by destroying the biological character of the phenomena“.

In the same way, the observed paces in a marathon are far from constant at an average speed. The best marathons are run with an asymmetric distribution of paces [6].

Nowadays, it is possible to test the hypothesis that the best marathon performance is run with a variable pace and to challenge the paradigm of a constant pace. Statistically, a race is run with an oscillating pace between extreme values that are inter-played by optimizing fuel, recovery, and avoiding VO2 and heart rate drift. Using mathematical statistics, we aim to show that an asymmetric distribution is optimal for marathon running, to delve deeper into the pace distribution, and to find the exact outline of the optimal pacing signature by analysing the official best world marathon performances by Dennis Kimoto (Berlin 2014) and Eliud Kipchoge (Monza 2017, Berlin 2018, 2019, Vienna 2019, and the Tokyo Olympics Games, 2021). Furthermore, previous marathon studies reported, also run in actual conditions, that physiological oscillations were the subjacent of real pace variations, even when the marathon pace is planned to be constant [6].

The Berlin marathons were official competitions and are official world records. While Monza 2017 and Vienna 2019 were exhibition marathons accomplished with rotating pacers, an electric pace vehicle, a laser beam projecting the ideal position on the road and ideal conditions. At Vienna, Kipchoge ran at a consistent average pace of 2:50 min per km (4:33.5 min per mile) and was 11 s ahead of schedule halfway through the marathon. He even accelerated in the final kilometre. Given that the best marathon performances, including the Olympics, were run by a single man (Eliud Kipchoge), this shows the possibility of adding a new dimension of running performance beyond the chronometer and proposing a qualitative way of optimizing the physiological capacity for running a marathon at 21 km/h. Our approach represents a powerful possibility for the marathon runner to validate the optimal aspects of his/her performance. However, before exploring the possibilities of providing feedback to a marathon runner, we must test the consistency of such a model in Eliud Kipchoge, who is purported to have a robust “performance template“. They have shown this to be primarily related to the increased confidence that the distance in question can be completed without unreasonable levels of exertion or injury [7]. This can apply to real-world conditions with the aid of mathematical modelling and wearable technology, as also already proposed in the journal Nature by the physicist Emig Thorsten, see the Ref. [2].

## 2. Materials and Methods

Our data consists of six marathon races: Berlin 2014, Monza 2017, Berlin 2018, Berlin 2019, Vienna 2019, and Tokyo 2021. They were performed by Dennis Kimetto, D.K. (Berlin 2014), Eliud Kipchoge, E.K. (Monza 2017, Berlin 2018, Vienna 2019 and Tokyo 2021), and Kenenise Bekele, K.B. (Berlin 2019).

For a given marathon, our data were collected from http://run.hwinter.de, accessed on 1 September 2021).

The original dataset includes marathons that provide timing data for 1km race segments (plus the final 41–42,195 km segment); the requirement for 1 km segments is based on the need to track changes in pacing during different stages of the marathon. The accuracy of the data is, then, higher than those collected by strava from the individual GPS measurement which depend on GPS models (between 0.47 and 1.65%, see the Ref. [8]). Thus, we will assume our data consist of a sequence, denoted by $\left(p\right)={\left(p_{i}\right)}_{1\leq i\leq 42}$, where $p_{i}$ is the pace, that is, the duration measured in s, taken to cover the *i*th km, all values being integers.

Following standard notations from the field of probability and statistics, if this sequence is arranged in order of magnitude and rewritten as

(1) $$p_{\left(1\right)}\leq \cdots \leq p_{\left(42\right)},$$

then $p_{\left(i\right)}$, for 1≤i≤42, it is called the *i*th order statistic (see §11.4 in the Ref. [9], or §1.1 in the Ref. [10]).

We call *average pace* the real number $\bar{p}$ computed from the sequence by the two equivalent relations

(2) $$\bar{p}=\frac{1}{42}\sum_{i=1}^{42}p_{i},\;\sum_{i=1}^{42}\left(p_{i}-\bar{p}\right)=0.$$

It is important to note that this value may differ slightly from what can be called the *official average pace* obtained by dividing the official total time by the distance 42.195 km. In our data, this only occurs for Vienna 2019. The resulting difference may, as usual, come from the phenomenon of rounding error, with the result that a total of rounded numbers is not equal to the rounded version of the original total. Furthermore, in our case, a rather precise value of the sum of the paces (minus the time taken to run the last 195 m) is known as the total time. The difference may also, in the case of a marathon, be accounted for, at least partially, by a very fast or slow pace during the last 195 m, that are not included in our measurements.

Since our aim is to provide computational methods for big data that have more available, we will therefore describe the stages of a statistical study starting after the sequence ${\left(p_{i}\right)}_{1\leq i\leq 42}$ has been produced.

The standard deviation σ=σ(p) of the pace sequence is defined to be

(3) $$\sigma^{2}=\frac{1}{42}\sum_{i=1}^{42}{\left(p_{i}-\bar{p}\right)}^{2}.$$

A coefficient of variation used extensively is Karl Pearson’s dimensionless coefficient of variation given by

(4) $$V=100\;\frac{\sigma }{\bar{p}},$$

and is one of the the standards for quantifying the relative pace variation.

In our study, a fundamental role will be played by the skewness coefficient

(5) $$\gamma_{1}=\frac{\sum_{i=1}^{42}{\left(p_{i}-\bar{p}\right)}^{3}/42}{{\left\{\sum_{i=1}^{42}{\left(p_{i}-\bar{p}\right)}^{2}/42\right\}}^{3/2}},$$

see formula (3.89) in the Ref. [9].

Computations from our data are summarized in Table 1.

The races are not listed chronologically, but according to the performance and the sign of $\gamma_{1}$.

It might seem, in view of the small values of *V* that the two performances, Vienna 2019 and Monza 2017, approached the uniform pace strategy. We will see, in fact, that these two performances and neither the other four marathon were run uniformly. This is discussed in the next paragraph.

## 3. Results

### 3.1. The Pace Is Not Uniform

As in everyday language, uniformity of the pace will mean for us that the race is run at an approximately constant pace. Mathematically, the sequence (p) is, in this case, a constant, or a sample of measurements drawn from a distribution highly concentrated around the average pace, for example, a normal distribution with mean $\bar{p}$ and very small variance.

Non-uniformity of the pace can be measured by the range of the observed pace distribution, that is, the difference between the greatest and smallest values observed, given by

(6) $$r_{1}=p_{\left(42\right)}-p_{\left(1\right)}.$$

Non-uniformity of the pace may also be measured by the difference between the fastest and the slowest 10 km,

$$r_{10}=\sum_{i=1}^{10}\left\{p_{\left(43-i\right)}-p_{\left(i\right)}\right\},$$

or by the difference between the fastest and the slowest half-marathon

$$r_{21}=\sum_{i=1}^{21}\left\{p_{\left(43-i\right)}-p_{\left(i\right)}\right\}=\left(p_{\left(22\right)}+p_{\left(23\right)}+\cdots p_{\left(42\right)}\right)-\left(p_{\left(1\right)}+p_{\left(2\right)}+\cdots +p_{\left(21\right)}\right).$$

Note that the well-known negative split is given by formula

$$r_{21}^{\prime }=\sum_{i=1}^{21}\left\{p_{43-i}-p_{i}\right\}=\left(p_{22}+p_{23}+\cdots p_{42}\right)-\left(p_{1}+p_{2}+\cdots +p_{21}\right),$$

so that the sum is calculated from paces ordered chronologically, whereas our sums in $r_{21}$ are performed after reordering the paces. Our ranges are known in the field of mathematical statistics as linear rank statistics.

The ratios

(7) $$V_{1}=\frac{r_{1}}{\bar{p}},\;V_{10}=\frac{r_{10}}{10\bar{p}},V_{21}=\frac{r_{10}}{21\bar{p}}$$

are the corresponding variation coefficients for 1, 10, and 21 km. The values are given in Table 2.

In view of this table, it is apparent that none of the six races manifests a range attributable to random fluctuations about an average target speed. In other words, none was run at an approximately constant pace, in spite of the fact that two of them, Monza 2017 and Vienna 2019 were planned to be run at a constant pace. The smallest ranges are achieved (as expected at Monza) at races designed to be run at a constant pace. As a matter of fact, 8 s, even for a non-elite runner, is a significant time-difference for a single kilometre, as well as 43 s for 10 km.

The most spectacular variation occurred for Tokyo 2021’s $r_{21}$, with more than 4 min between the fastest and the slowest half-marathon in the same race.

### 3.2. The Pace Is Not Symmetric

Once the spread of the pace has been shown to be significant, it is legitimate to raise the issue of its shape, in particular, its symmetry or lack of symmetry.

**Figure 1:**
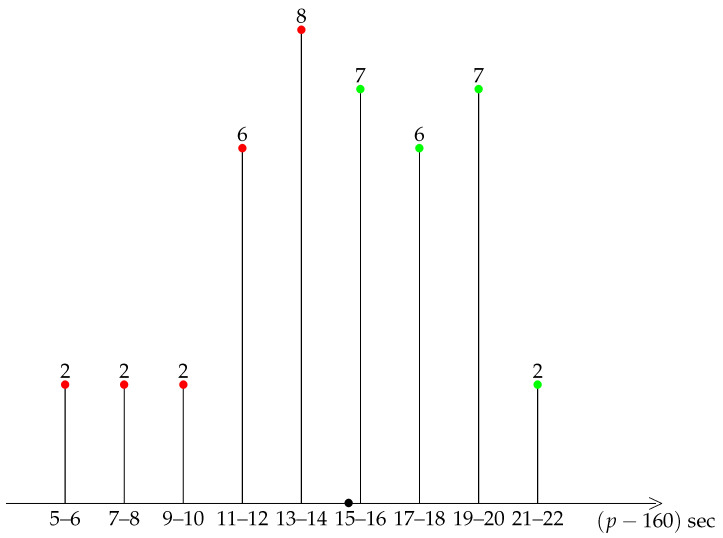
Berlin 2014. Frequency of paces. For instance 8 km were run at the pace 173 or 174 s. Slow (resp. fast) km in green (resp. red). The black bullet on the *p* axis marks the average pace.

**Figure 2:**
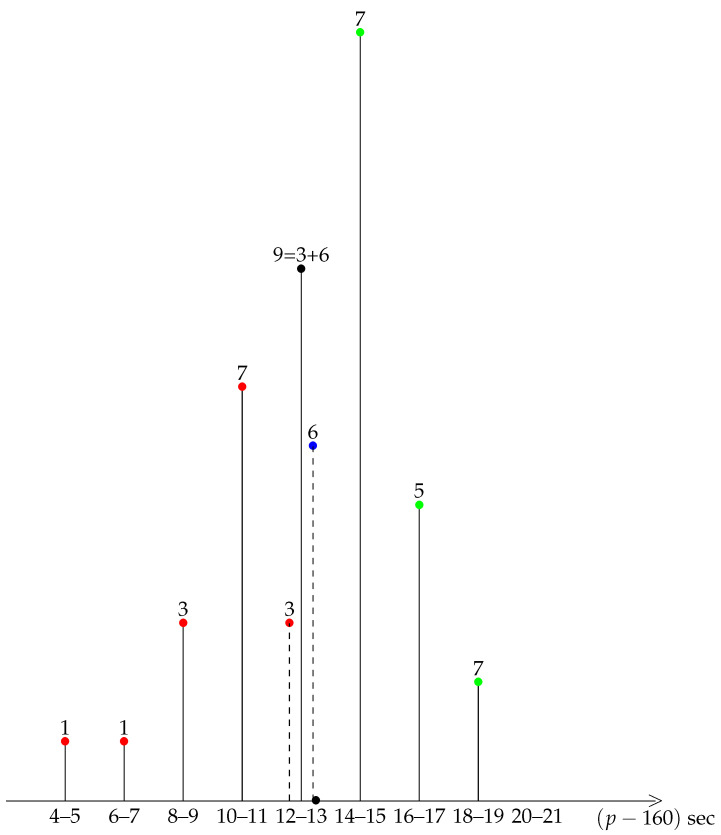
Berlin 2019. Slow (resp. medium, fast) km in green (resp. blue, red). The 12–13 class is decomposed into two subclasses, 3 fast and and 6 medium km.

**Figure 3:**
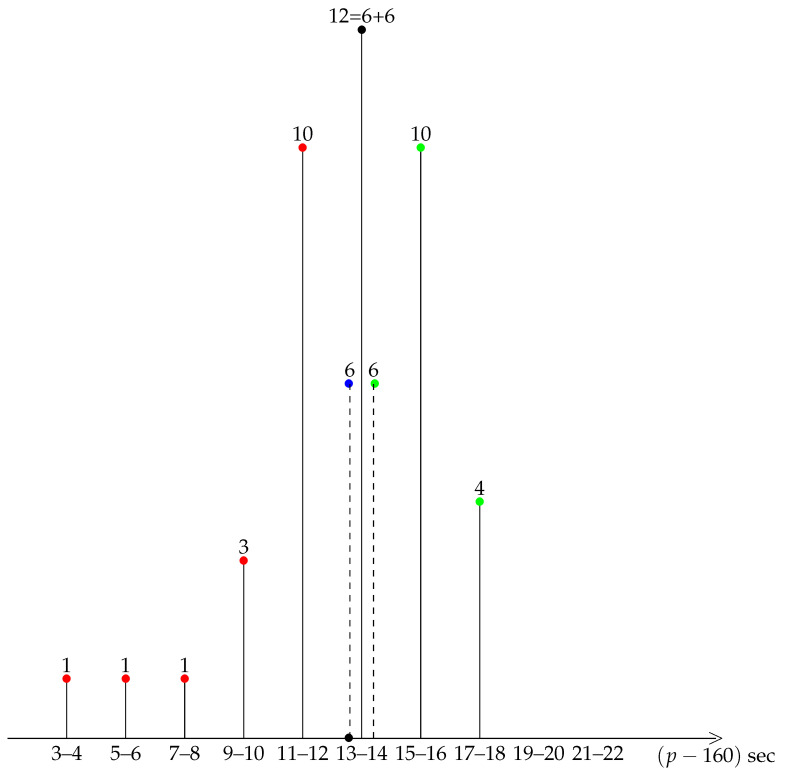
Berlin 2018. Slow (resp. medium, fast) km in green (resp. blue, red). The 12–13 class is decomposed into two subclasses, 6 medium and 6 slow km.

**Figure 4:**
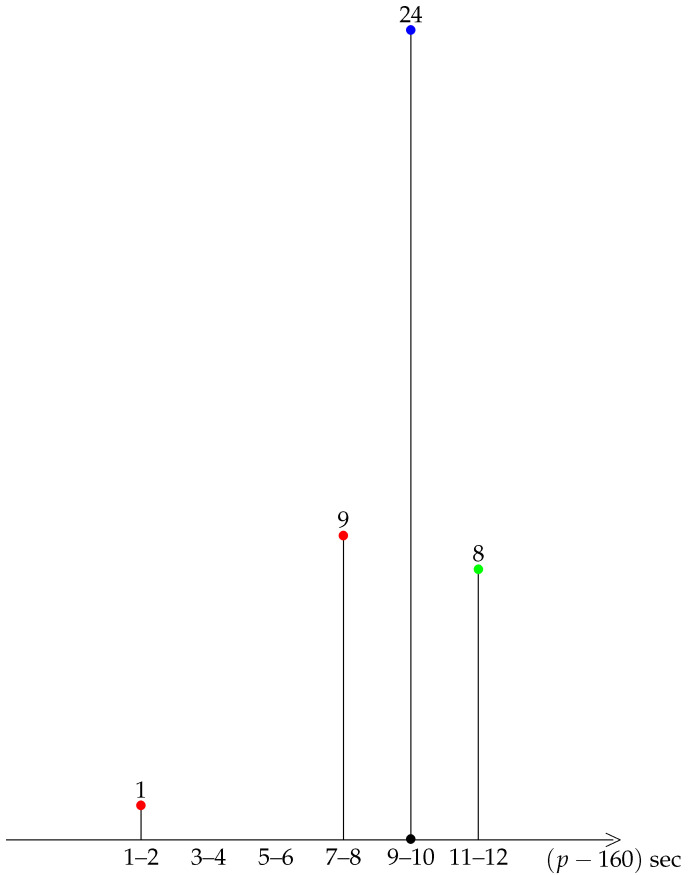
Vienna 2019.

**Figure 5:**
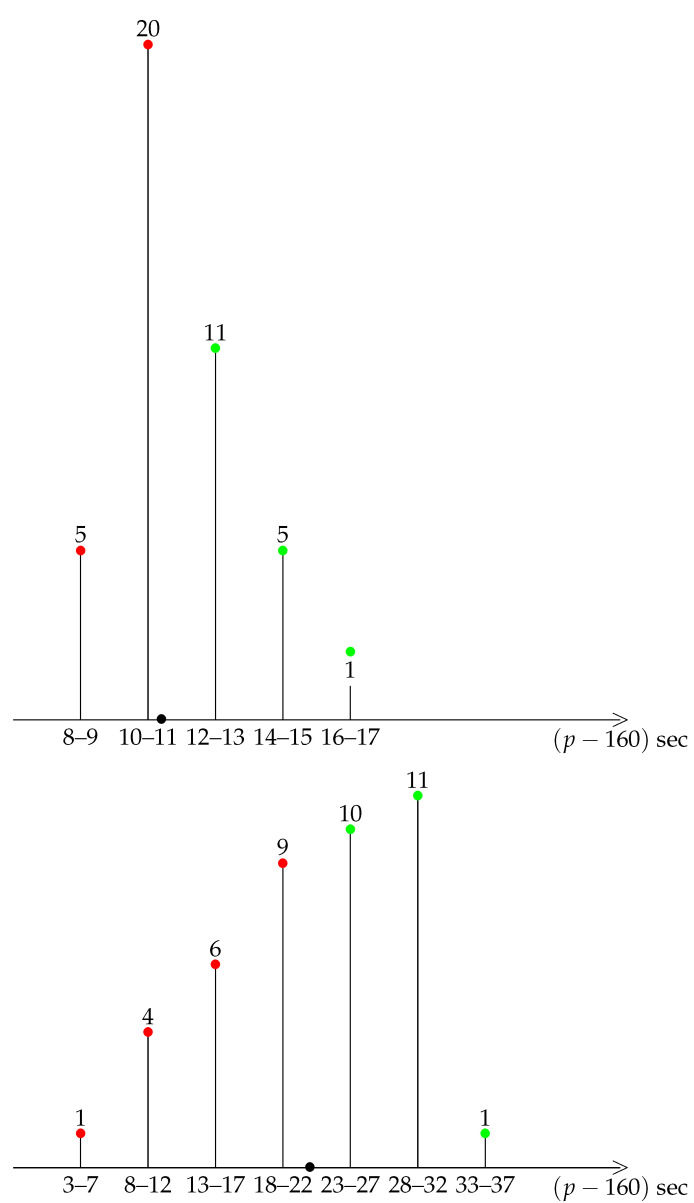
Monza 2017 and Tokyo 2021. Number of slow (resp. fast) km in green (resp. red). The black bullet on the *p* axis marks the average pace.

A first insight into the general shape of our distribution is provided by a diagrammatic representation of the data, see the bar charts of grouped data in Figure 1, Figure 2, Figure 3, Figure 4 and Figure 5.

A striking feature shared by the six races is that they belong to the class of moderately asymmetric bell-curves (see the Ref. [11], Section 4.20 and Figure 4.7 pp. 83–87): the class frequencies present one maximum, and fall rapidly on one side of the maximum when compared to the other.

Note that for asymmetric distributions with negative skewness, the median value is usually greater than the mean, which means in our case that the runner will run more than half of the race with a pace above the average pace value, making the race more comfortable. We discussed this aspect in the Ref. [6].

Statistically, we can assess that our sequences of paces do not behave as random samples from populations (in the sense used in mathematical statistics, see Section 1.1 and Chapter 9 in the Ref. [9]) with symmetric, for example, normal, distributions. In this case, $\gamma_{1}$ would be close to zero, up to sampling fluctuations. Large positive or negative values of $\gamma_{1}$ indicate a departure from normality, or symmetry.

To this end, we make use of Zar’s statistic denoted by *z* (see pp. 115–116 in the Ref. [12], or Equations I.23 p. 21, (4.2)–(4.8) pp. 227–228 in [13], restated in Equations (8)–(14), to be applied with *n* = 42).

Formulas leading to Zar’s statistic are as follows.

(8) $$\begin{array}{c} A=\gamma_{1}\sqrt{\frac{\left(n+1)(n+3\right)}{6(n-2)}}, \end{array}$$

(9) $$\begin{array}{c} B=\frac{3\left(n^{2}+27n-70\right)\left(n+1\right)\left(n+3\right)}{\left(n-2)(n+5)(n+7)(n+9\right)}, \end{array}$$

(10) $$\begin{array}{c} C=\sqrt{2(B-1)}-1, \end{array}$$

(11) $$\begin{array}{c} D=\sqrt{C}, \end{array}$$

(12) $$\begin{array}{c} E=\frac{1}{\ln\sqrt{D}}, \end{array}$$

(13) $$\begin{array}{c} F=A\sqrt{\frac{C-1}{2}}, \end{array}$$

(14) $$\begin{array}{c} z=E\ln(F+\sqrt{F^{2}+1}). \end{array}$$

In the case of a normal distribution for (p), then *z*, computed from our $\gamma_{1}$, would approximately follow the distribution of a standard random normal variable, denoted by N(0,1).

For the six races, *z* takes values fairly, or even significantly different from zero. A positive skewness of the pace is associated with the failed performance of Monza 2017.

A negative skewness of the space is associated with the successful performances.

Furthermore, when the skewness is negative, we observe the final times are better resulting in a higher (in absolute value) skewness (see Table 3).

The skewness may also be measured in the way the range $r_{1}$ splits into a left range $\bar{p}-p_{\left(1\right)}$ (difference between the average pace and the fastest kilometre’s pace) and a right range $p_{\left(42\right)}-\bar{p}$ (difference between average pace and the slowest kilometre’s pace). The difference between these two ranges, say Δ2R, defined by

(15) $$\Delta 2R=\left(p_{\left(42\right)}-\bar{p}\right)-\left(\bar{p}-p_{\left(1\right)}\right)=p_{\left(42\right)}+p_{\left(1\right)}-2\bar{p},$$

can be seen as a measure of skewness.

Table 4 confirms that negative asymmetry is associated with a successful performance.

### 3.3. The Sign of Asymmetry Is Not Due to Random Sampling Fluctuations

Even though most distributions from the real world manifest asymmetry, we can show that the negative asymmetry observed on the six registered races cannot be explained by mathematical randomness, as shown by the following thought experiment (in the sense of Einstein’s *Gedankenexperiment*).

If it were the case, the observation of a race would be as frequent as that of what we call the *associated mirror-race*, say $p^{*}$, defined by one of the two equivalent equalities

(16) $$p_{i}^{*}=2\bar{p}-p_{i},\;\frac{p_{i}^{*}+p_{i}}{2}=\bar{p}\;\left(1\leq i\leq 42\right).$$

For example for Berlin 2018, where the average pace was $\bar{p}\approx 173$ sec, a kilometre run in 175 sec would be replaced in the mirror race by a kilometre run in 171 sec. The average of these two values is $\bar{p}=173$.

From the equalities

$$p_{i}^{*}-\bar{p}=-\left(p_{i}-\bar{p}\right)\;\left(1\leq i\leq 42\right)$$

and in view of Equations (2)–(5) we infer that a race and its mirror image have the same average pace (in other words they correspond to the same performance measured by the final time), and opposite asymmetries, as measured by $\gamma_{1}$. In the 2018 Berlin marathon, 16, 6, and 20 km were run at a pace smaller, equal to, or greater than the average $\bar{p}$. In other words, 16 km were ran fast, 6 km were medium, and 20 km were slow. In the mirror-race, 16 km would be slow, 6 km would remain medium, and 20 km would be fast. Firstly, it is clear that the actual race is more comfortable to run than the mirror race. Secondly, physiologically, whereas 20 slow km gives the possibility to recover energy for 16 fast km, it is not certain that 16 slow km would enable the runner to run 20 fast km, as shall be discussed below.

Remarkably, Berlin 2019 manifests a similar split of 15, 6, and 21 km (fast, medium, and slow km). Hence, the mirror race would also be much more difficult to perform in this case.

For Tokyo 2021, Berlin 2014, and Monza 2017, the average pace is distinct enough from an integer value, so that each kilometre can be labelled as fast/difficult (red) or slow/easy (green), see Figure 1 and Figure 5. For Tokyo 2021 and Berlin 2014, there were more easy kilometres than difficult kilometres, so that running the mirror race would again be unreasonable. For Monza 2017, the situation is the opposite, and there were more difficult than easy km. Therefore, the mirror race, with positive skewness, would have been easier to run.

For Vienna 2019, a cursory look at the bar chart suggests that the distribution can be seen as the mixture (see Section 1.2.14 in the Ref. [14]) of two independent distributions. The first one, represented by the right component of the graph, is rather symmetric and concentrated around the mean value $\bar{p}\approx 170$ s. The second one is reduced to that of one value $p_{\left(1\right)}=p_{42}=161$ sec, a very fast pace during the last kilometre. The negative asymmetry is mainly due to this last kilometre, that is, the fact that the runner had an excessive amount of energy at the end of the race. The mirror race would consist of the superposition of the same symmetric distribution centred about the average pace, and of a very high value corresponding to a very slow kilometre, which is unrealistic.

## 4. Discussion

The future of marathon training will focus on the qualitative aspects, versus only focusing on the quantitative data. This is certainly a step in the right direction, as it was Kipchoge’s confidence that allowed him to break the sub-2 h marathon barrier. Advances in wearable sensor technology have enabled real-time measurement of physiological data during exercise [2]. Future directions in training are going to be about encouraging the marathon runner. To achieve this, we must provide ways of improving satisfaction regarding training progress and with the feeling that it has been optimized. We are testing the hypothesis that the ideal race, with the world’s best marathon runners, can have several degrees of optimization. This can be observed in Kipchoge’s last five marathons, including the exhibition marathons, where the paradigm of a perfect constant pace was applied. In the official marathons (Berlin), the pace was not constant and run with other competitors; these races are considered as the best performances ever ran.

The best races are run with a negative asymmetry of pace distribution. This means that more time is spent running below Vmarathon and that the pace is variable, never constant. Our recent publication about the asymmetric characteristics of marathon pace in elite marathon runners were not random, but well-founded on mathematically demonstrated principles. Estimating the optimal marathon pace should be done as a consequence of optimal physiological homeostasis. Our results showed how to obtain a true individual distribution shape, for instance, concave or bathtub, by using the negative asymmetry of pace distribution. We reported an optimal value for the negative asymmetry, showing how this asymmetric distribution results from an oscillating pattern and the interaction between extreme values.

### 4.1. The Pace Is Variable, Not Uniform

Publications showing that pace is non-uniform are in agreement with prior studies of short-distance competitions. Indeed, variability in pacing has been studied in short- and middle-distance running (e.g., 3000 m to 10 km) (see the Refs. [15,16,17,18,19,20]).

These studies have focused on the influence of variability of pacing on metabolic and performance parameters. However, the Ref. [21], reported that elite marathon runners had few changes in pace, suggesting low speed variability. More recently, the sub-2 h marathon attempts in Monza (2017) and Vienna (2019) were based on the belief that constant speed is the best way of running. These ideas were derived from the notion that an optimal pace is non-variable, according to the seminal model of the Ref. [3].

Recreational marathon runners adopt the same paradigm of constant speed by running with a pace-group leader as provided by the marathon organizers. This blindly reinforces the idealistic paradigm of constant speed, distorting the practice of running. However, considering the physiological limitations (glycogen availability) means that runners must choose the ideal pace, allowing them to achieve the optimal performance without famously “hitting the wall“ or slowing down from extreme fatigue 10 km or less before the finish line (see the Ref. [22]).

We must underline that negative pacing can help runners to achieve better results (both recreational and professional). However, a great majority of runners just want to complete a marathon or enjoy running, without focusing too much on optimizing their pacing. Therefore, for them, even pacing is easier to follow and control. Indeed, for both elite and recreational runners, planning variable pacing is not practical. The pacing is mainly controlled by CNS and it is highly automated. Therefore, any change in pacing can be hard to achieve, resulting from runners either slowing down or speeding up too much, particularly if they are less experienced runners. Please elaborate some more, and possibly add this as a limitation of this study. In addition, speeding up and slowing down requires additional muscle force to be exerted, thus spending extra energy even if it allows for elevation of the average pace. We still have to check the difference in energy spent by continuous energy cost measurements between variable and even pace race, at least in a real half-marathon race.

The non-arbitrary sign of asymmetry, which cannot be accounted for by sampling fluctuations in speed but by physiology, is explained by the “mirror race“. It is important to understand that a runner cannot recover lost time in the first part of a marathon (as in Monza) using their speed reserve (see the Refs. [23,24]). Recall that speed reserve is defined as the difference between the velocity at VO2max and the maximal speed during a sprint or a 1000 m distance (see the Ref. [25]).

Variable pace running focuses the optimization of energy. These concepts were developed by AV Hill over 100 years ago with his papers on muscular exercise, lactic acid, the supply and utilization of oxygen, all helping to establish the concept of “an-aerobic“ energy production during exercise, with oxidative restoration in recovery (see the Ref. [26]); it is unlikely that he thought humans should run at a constant pace to achieve athletic records. Ironically, ever since Hill’s time, schools often still teach students to run at a strictly constant pace, rather than to self-pace according to the rate of perception of exhaustion. It has been proven that humans are natural runners and capable of accurately self-pacing our accelerations and speed variations at three levels of intensity (soft, medium, and hard accelerations) (see the Ref. [27]). Even if control of speed, and thus power output, is voluntary (see the Ref. [28]), the physiological signals that athletes receive by estimating their abilities to sustain any instantaneously chosen speed have yet to be to be elucidated. Computing the critical speed from a personal best has shown, in the same way as for VO2max, that all the elite marathon runners could run very close to their critical speed (90–98%) [29,30].

Furthermore, it has been shown that critical speed can be accurately estimated from a non-exhaustive self-pace run [31]. Even if these two paradigms of exercise (self-pace or constant-load model) have different limitations (see the Ref. [32]), the self-pace model has allowed new insights into the optimization of energy transformation (see the Ref. [33]). Energy transformation during exercise is thought to be variable; this allows for self-regulation so that feed-forward or anticipatory regulation remains a critical signal to preserve homeostasis and to avoid cellular catastrophe (see the Ref. [34]). The Ref. [33] showed that it is possible to consider the time required for task completion, which can explain why power output (or speed) and skeletal muscle recruitment (and hence VO2) in self-pace trials are almost immediately reduced in exercise, only to return to near-optimal values during the “end spurt” (see the Ref. [35]). Examining the pacing signatures of the two best marathon performances and, given that the same human has achieved these results, offers us the unique possibility to compare these performances and further understand how the limits of human physiology were elevated. In addition, we could also apply this novel research to the over 9 million marathon runners who crossed the finish line in 2019.

### 4.2. The Pace Is Not Symmetric, and the Sign of Asymmetry Is Explained via a “Mirror Race“, by Physiology, and Not by Sampling Fluctuations

Independent of the race or the runner, the pace is never symmetric, implying that the central tendency and deviation do not entirely characterize the pacing signature. A negative asymmetry of pace distribution signifies a faster race and the higher the absolute value of the pacing asymmetry is, the better the performance is. Indeed, according to our hypothesis, our results show that for each of these high-level races, we could give the general shape of the data distribution. Independent of the marathons studied, we always obtained an asymmetric shape. Interestingly, we showed that the asymmetries for the two exhibition marathons, were different: negative for Vienna (the first 2-sub-hour marathon race (1 h 59 min 40 s and γ=−2.50) and positive in Monza (2 h 00 min 25 s and γ=+0.56). Furthermore, we showed Vienna was comprised of two independent distributions, therefore it was really two different races: one was constant for 40 km, and the other, also constant, but at a much higher speed. Remarkably, in the Vienna attempt, Kipchoge had a remarkable “end spurt“ in the final kilometer, helping him to break the 2 h barrier, and thus creating an artificial asymmetry. Similarly in Tokyo, after running a very slow first half-marathon while attempting to help his compatriot, Kipchoge finished the last 10 km extremely quickly. The marathon pace (or speed) frequency distribution manifests two remarkable features: non-uniformity, and skewness of the pace which has the particularity of being negative. This is a condition of comprehending a good performance, that is, the shortest time possible without excessive suffering. A U-shaped curve allows the complete distribution of pacing. By now, pacing profiles and tactical behaviors of elite runners have been shown to be a U-shape from the 1500 to the 10,000 m (Casado et al., 2020). This U-shape is characterized by a fast start, a slowing down during the middle part of the race, and a fast finish, characterizing world record performances (see the Ref. [36]).

Although a parabolic J-shaped pacing profile (in which the “fast-start“ is faster than the middle part of the race but is slower than the end spurt) is observed in many championship races [36], we showed that in the marathon, the best race is in U shape, which induced a negative speed asymmetric distribution measured by Δ2R. Here, we reported the shape distribution of the entire marathon, and we have formalized the role of the extreme values and oscillations between them. In these world record performances, we show that non-uniformity of a sequence of measurement means that they do not arise from a population highly concentrated around a sample value as a normal distribution with a very slight variance.

We also showed how the difference in extreme values distributions allows a characterization of the optimal marathon as negatively asymmetric in the sense that the fast-km pace frequency decay occurs more progressively than the slower km pace. Analysing the “big data“ of marathons reveals that foremost, the individual experience of training and running a marathon offers personal gratification, whether or not they achieve their target of finishing less than 4 h 21 min 03 s or 4 h 48 min 45 s (the average male and female marathon performances in 2018, respectively). Endurance running has never reached this level of popularity, especially among women. Marathon running is truly human by nature (see the Ref. [37]). In this fundamental article, the authors showed how evolution has not only shaped the human for long-distance running, but has also conditioned the human brain to enjoy this type of physical activity through the development of endorphin release and mood-elevating neuroendocrine mechanisms. When humans run, we are balanced between a quest for performance and “the minimization of effort“, which is defined as the process that aims to achieve the most cost-effective behaviour based on our perceptions (see the Ref. [38]). Energy is the ability to produce physical action and effort is the cortical brain activity associated with the initiation or maintenance of a behaviour. The brain constructs our perceptions based not only on the current physical effort, but also on previous similar experiences, motivation, awareness, and effects (see the Ref. [39]). Therefore, our theory of a mirror race, that may or may not be possible to run, shows that an optimal marathon performance must be performed without suffering, and hence with variable pace.

### 4.3. Some Mathematical Speed Functions as the Bathtub with Negatively Skewed Pace Distributions

Let us show that the foregoing results are, obtained via calculus, in agreement with some already identified running strategies, especially faster start and finish that are observed even in the traditional (even-pacing) approach to record-setting results.

Assume that q∈(0,1/2). Typically, *q* will represent the proportion of distance run fast during a marathon. For instance the runner runs 42q km fast at pace $p_{1}$, and 42(1−q) km slowly at a pace $p_{2}>p_{1}$. The bar chart of the pace sequence corresponds to the increasing function, say ϕ, defined by

$$42q=\phi \left(p_{1}\right)<\phi \left(p_{2}\right)=42\left(1-q\right)\;\left(p_{1}<p_{2}\right).$$

**Figure 6:**
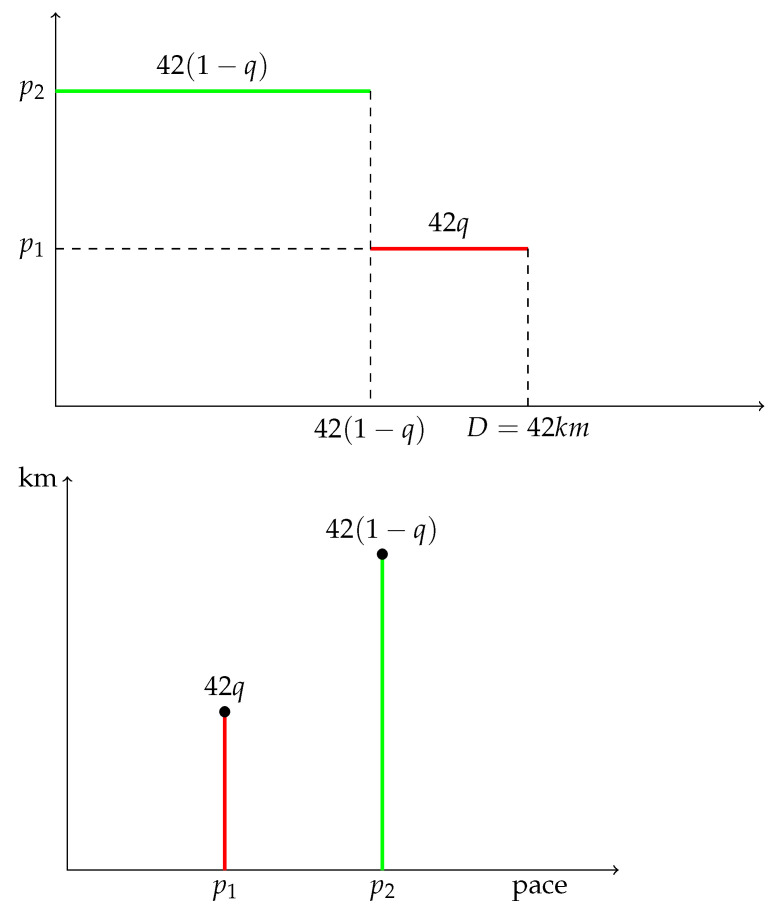
A race with two paces, negative asymmetry.

The bar chart of the pace distribution ϕ, given by Figure 6, is that of a Bernoulli distribution with skewness coefficient

$$\gamma_{1}=\frac{2q-1}{\sqrt{q(1-q)}}<0.$$

Let us generalize this fundamental example. Let s∈[0,42] denote the curvilinear abcissa, that is, the distance from the start line. The instantaneous pace at *s* is denoted by p(s).

The bar charts of the pace sequence considered up to now will be replaced by a continuous curve, the density function p↦ϕ(p), defined as follows. The infinitesimal distance ds=ϕ(p)dp represents the distance spent by the runner at the pace ranging from *p* to p+dp. In accordance with the preceding example, we assume that ϕ is increasing. The derivative of s↦p(s) is $p^{\prime }\left(s\right)=dp/ds$. The preceding equalities lead to

(17) $$\frac{dp}{ds}=\frac{1}{\phi (p)},\;\phi \left(p\right)dp=ds,\;\Phi \left(p\right)=s,$$

where Φ(p) is such that $\Phi^{\prime }\left(p\right)=\phi \left(p\right)$. We obtain

(18) $$p\left(s\right)=\Phi^{-1}\left(s\right)$$

as the pace function. Note that equation $p^{\prime }\left(s\right)=\phi \left(p\right)$ and the fact that ϕ is assumed to be an increasing function of *s*, which is itself increasing with *p*, imply that $p^{\prime }$ is decreasing, thus *p* is concave. Up to a constant factor the speed function is given by v(s)=1/p(s), so that

(19) $$v^{\prime \prime }\left(s\right)=-\frac{p^{\prime \prime }}{p^{2}}+2\frac{{\left(p^{\prime }\right)}^{2}}{p^{3}}>0,$$

and *v* is an increasing convex function.

Conversely, every increasing speed function s↦v(s) gives rise to a concave decreasing function s↦p(s) (invert the roles of *v* and *p* in Equation (19)) and to a density function $\phi \left(p\right)=1/p^{\prime }\left(s\right))$ that is increasing, as the inverse of a decreasing function.

Let us make these formulas explicit in the case

(20) $$\phi \left(p\right)=p^{\alpha },\;\left(0<\alpha <1\right)$$

so that ϕ is a beta distribution, with negative skewness [14] Chapter. Then up to multiplicative factors, $\Phi \left(p\right)=p^{\alpha +1}$,

(21) $$p=\Phi^{\left(-1\right)}\left(s\right)=s^{\frac{1}{\alpha +1}}=s^{\beta }\;\left(0<\beta <1\right)$$

Since $\gamma_{1}\left(p\right)$ is invariant under affine transformations, we can set

(22) $$p\left(s\right)=C_{1}+C_{2}s^{\beta },\;v_{0}\left(s\right)=\frac{1}{C_{1}+C_{2}s^{\beta }}$$

These computations lead to rather unrealistic pace strategies. But keep in mind that our aim was only to produce some pace distribution with negative skewness.

From the speed function in Equation (22), we can build two speed functions that will lead to the same density function ϕ:

(23) $$v_{1}\left(s\right)=\left\{\begin{array}{cc} v_{0}\left(s\right), & \mathrm{for}\;0<s<T,\\ v_{2}\left(s\right)=v_{0}\left(s-T\right), & \mathrm{for}\;T<s<2T \end{array}\right.,$$

and

(24) $$\;v_{2}\left(s\right)=\left\{\begin{array}{cc} v(s), & \mathrm{for}\;0<s<T,\\ v_{2}\left(s\right)=v\left(2T-s\right), & \mathrm{for}\;T<s<2T \end{array}\right.$$

**Figure 7:**
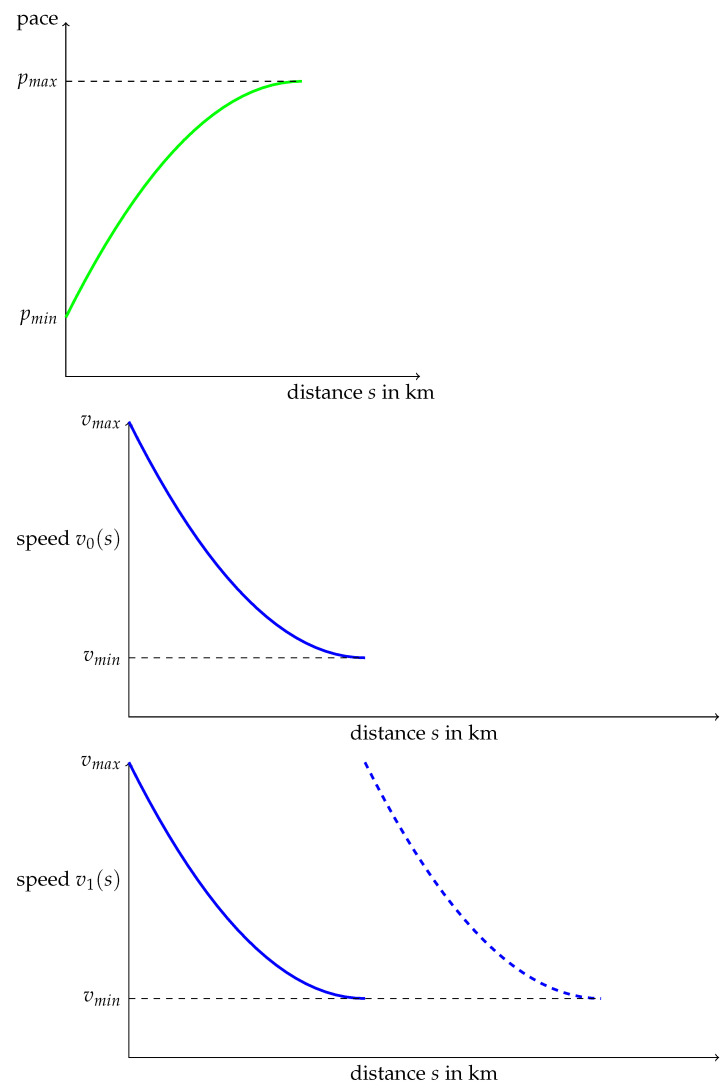
A concave pace function on an elementary time stretch, a convex speed function $v_{0}$, for instance (22) on an elementary time stretch, and a speed function $v_{1}$ with the same frequencies and asymmetry as $v_{0}$.

**Figure 8:**
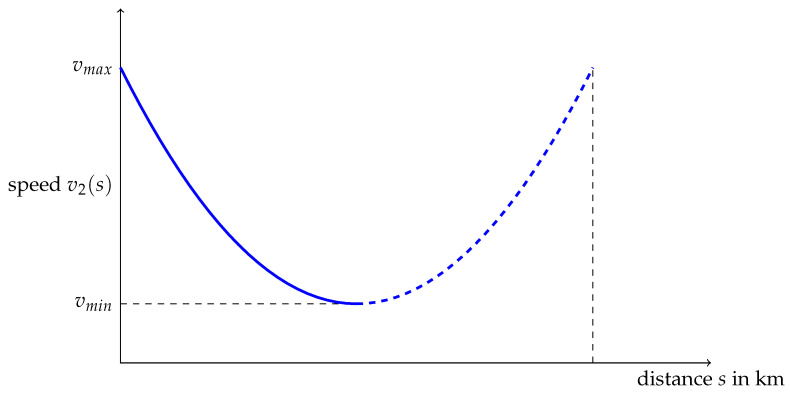
Bathtub curve function $v_{2}$ with the same frequencies ans asymmetry as $v_{0}$.

The graphs of $v_{1}$ and $v_{2}$ are drawn in Figure 7 and Figure 8, the dashed parts being those which were added. Periodicity and symmetry, used to build $v_{1}$ and $v_{2}$ respectively, can be used an arbitrary number of times.

From a mathematical point of view, a running strategy can be outlined as follows: First, divide the race into sub-races, called “pace-cells“, and for each of these cells, select a speed function as $v_{0}$, $v_{1}$ or $v_{2}$. Provided these functions are built from the same elementary function as $v_{0}$, the global skewness will be that of $v_{0}$.

Figure 7 can be associated with an oscillator as a clock with frictions, receiving regular impulses, see the Ref. [40], Section III.4.2.

The theory of oscillators seems well-suited for providing models for oscillating functions. Our analogy is not based on purely mathematical similarities, but is a consequence of our results. The interplay between extreme (small and large) values that we have unveiled, corresponds, for oscillators, to the well-known restoring forces. Indeed, Claude Bernard already pointed out that all biological systems are oscillators, in the sense that the value of a variable remaining constant at its mean value is never observed, that is to say biological systems are oscillators. Our statistical analysis illustrates this fact concerning the world’s best marathon runners.

### 4.4. Oscillations between Extremes Values Allow the Interplay between Aerobic and Anaerobic Metabolisms, Optimizing the Recovery of Energy

We proposed to focus on a time series generated by the oscillating pattern of the damped clock discussed in the Ref. [40]. Remarkably, when human and other mammals run, the body’s complex system of muscles, tendons and ligament springs behaves like a single linear spring (“leg spring“). A simple spring-mass model, comprising a single linear leg spring and a mass equivalent to the animal, has been shown to describe the mechanisms of running remarkably well (see the Ref. [41]). Hence, the stride can be considered as a damped clock. We have shown that the first sub-2-h attempt (Vienna) was not optimal. Therefore, by considering the pacing oscillations between extremes values according to the athlete’s physiology, these world record performances could be run faster, even the 1 h 50 min marathon as predicted by [42]; but only when the method for predicting the record is based on physiology. Indeed, currently it is thought that running a marathon at the fastest speed possible appears to be regulated by the rate of aerobic metabolism (i.e., marathon oxygen uptake) of a limited amount of carbohydrate energy (i.e., muscle glycogen and blood glucose) and the pace that can be maintained without developing hyperthermia.

However, to oscillate between extreme pace values, there is a need to have a high range of speed incompatible with monotonous training [43,44]. Indeed, our results confirmed that the best performance was achieved by an oscillating pattern between extreme paces.

Extreme value theory is used to predict the occurrence of rare events such as extreme floods, large insurance losses, stock market crashes, and human life expectancy. Indeed, some authors have already applied the extreme value theory to athletic events, using estimation methods involving moment method and maximum likelihood methods (MLE). Accurate estimations of future athletic records were accomplished using both methods. Hence, an interesting question is: under the present knowledge of training, materials (shoes, suits and equipment), and anti-doping regulations, by how much further could athletes possibly exceed current world records in the near future? We used the extreme value theory dealing with the issues of extremes without considering them to be independent because we took into account (as stated by Hill), the “fatigue factor“.

Running a marathon between extreme values, training must use the so-called “polarized training“ emphasizing low and high intensity, rather than medium intensity (see the Refs. [43,45]). A recent study based on around 14,000 individuals with more than 1.6 million exercise sessions containing duration and distance, and with a total distance of 20 million km showed that the analysis of individual long-term training protocols leads to a wide spectrum of physiological responses [2]. This study on big data confirms the concept of “polarized training“, which involves running a wide range of speeds; these techniques are currently used by elite athletes and have been shown to be the most efficient training methods, resulting in the greatest improvements in the key variables of endurance performance in well-trained endurance athletes.

## 5. Conclusions

We analysed the three best real-world marathon performances ever ran to the hypothesis that Kipchoge optimizes his pacing in relation to his aerobic and anaerobic power and endurance. The conclusions from this could be a source of inspiration for the over 9 million marathon runners who crossed the finish line in 2018 (Esther Fleming, https://www.sidmartinbio.org/how-many-runners-are-there/, (accessed on 1 September 2021). Using mathematics and physiology, we opened up new perspectives on how optimize endurance and power in a marathon. The pacing strategy of elite marathon runners is to start fast and then to recover by running just below their average pace for 2/3 of the racing distance, generating a negative asymmetry, that is, a median speed below the average. This “lazy“ race confirms AV Hills’ discoveries about the concept of “an-aerobic“ energy production during exercise, with oxidative restoration in recovery being a factor determining the variation of speed with distance.

However, our aim was not to replace a coach’s advice, but only to show that the future of the best performances, as in world records, is perhaps to look to the variable pace shape.

## Figures and Tables

**Table 1:** Mean $\bar{p}$, standard deviation σ, coefficient of variation *V* and skewness $\gamma_{1}$ for the six marathons.

Race	Total Time	$\bar{p}$	σ	*V*	$\gamma_{1}$
Tokyo 2021 (E. K.)	2 h 08 min 38 s	182.4 s	7.2	4%	− 0.31
Berlin 2014 (D.K)	2 h 2 min 57 s	174.8	4.1	2%	− 0.47
Berlin 2019 (K.B)	2 h 01 min 41 s	173.0	3.0	2%	− 0.55
Berlin 2018 (E.K)	2 h 01 min 39 s	173.1	3.0	2%	− 0.82
Vienna 2019 (E.K)	1 h 59 min 40 s	169.7	1.9	1%	− 2.32
Monza 2017 (E.K)	2 h 00 min 25 s	171.2	1.8	1%	+0.56

**Table 2:** Absolute and relative ranges computed from order statistics.

Race	$\min=p_{\left(1\right)}$	$\max=p_{\left(42\right)}$	$r_{1}$	$r_{10}$	$r_{21}$	$V_{1}$	$V_{10}$	$V_{21}$
Tokyo 2021	167	194	27	189	254	15%	10%	7%
Berlin 2014	165	181	16	106	137	9%	6%	4%
Berlin 2019	165	179	14	76	98	8%	4%	3%
Berlin 2018	164	178	14	73	124	8%	4%	3%
Vienna 2019	161	172	11	43	43	6%	2%	1%
Monza 2017	168	176	8	43	58	5%	3%	2%

**Table 3:** Zar’s statistic *z*. The *p*-value of *z* is P(N(0,1)>z) if z>0, or P(N(0,1)<z) if z<0.

Race	$\gamma_{1}$	*z*	*p*-Value of *z*
Tokyo 2021	−0.31	−0.92	18%
Berlin 2014	−0.47	−1.36	9%
Berlin 2019	−0.55	−1.50	7%
Berlin 2018	−0.82	−2.25	1%
Vienna 2019	−2.32	−4.79	$<10^{-6}\%$
Monza 2017	0.56	2.07	2%

**Table 4:** Skewness measured by coefficient Δ2R.

Race	$p_{\left(42\right)}-p_{\left(1\right)}$	$\bar{p}-p_{\left(1\right)}$	$p_{\left(42\right)}-\bar{p}$	Δ2R
Tokyo 2021	27	15.4	11.6	−3.8
Berlin 2014	16	9.8	6.2	−3.6
Berlin 2018	14	9.1	4.9	−4.2
Vienna 2019	11	8.7	2.3	−5.4
Monza 2017	8	3.3	4.7	1.2

## Data Availability

https://labs.strava.com/, accessed on 1 January 2022.
